# Quantitative and Functional Characterization of the Hyper-Conserved Protein of *Prochlorococcus* and Marine *Synechococcus*

**DOI:** 10.1371/journal.pone.0109327

**Published:** 2014-10-31

**Authors:** Caroline E. Whidden, Katrina G. DeZeeuw, Jackie K. Zorz, Andrew P. Joy, David A. Barnett, Milo S. Johnson, Olga Zhaxybayeva, Amanda M. Cockshutt

**Affiliations:** 1 Department of Chemistry & Biochemistry, Mount Allison University, Sackville, NB, Canada; 2 Atlantic Cancer Research Institute, Moncton, NB, Canada; 3 Department of Biological Sciences, Dartmouth College, Hanover, New Hampshire, United States of America; 4 Department of Computer Science, Dartmouth College, Hanover, New Hampshire, United States of America; Hellas, Greece

## Abstract

A large fraction of any bacterial genome consists of hypothetical protein-coding open reading frames (ORFs). While most of these ORFs are present only in one or a few sequenced genomes, a few are conserved, often across large phylogenetic distances. Such conservation provides clues to likely uncharacterized cellular functions that need to be elucidated. Marine cyanobacteria from the *Prochlorococcus*/marine *Synechococcus* clade are dominant bacteria in oceanic waters and are significant contributors to global primary production. A Hyper Conserved Protein (PSHCP) of unknown function is 100% conserved at the amino acid level in genomes of *Prochlorococcus*/marine *Synechococcus*, but lacks homologs outside of this clade. In this study we investigated *Prochlorococcus marinus* strains MED4 and MIT 9313 and *Synechococcus* sp. strain WH 8102 for the transcription of the PSHCP gene using RT-Q-PCR, for the presence of the protein product through quantitative immunoblotting, and for the protein's binding partners in a pull down assay. Significant transcription of the gene was detected in all strains. The PSHCP protein content varied between 8±1 fmol and 26±9 fmol per ug total protein, depending on the strain. The 50 S ribosomal protein L2, the Photosystem I protein PsaD and the Ycf48-like protein were found associated with the PSHCP protein in all strains and not appreciably or at all in control experiments. We hypothesize that PSHCP is a protein associated with the ribosome, and is possibly involved in photosystem assembly.

## Introduction

Cyanobacteria are ubiquitous oxygenic phototrophs on Earth, colonizing all environments reached by solar light and largely responsible for the fifty percent of global net primary production that occurs in the world's oceans [1], [2]. Numerically dominating the oceanic waters are marine free-living unicellular picocyanobacteria from *Prochlorococcus* and Cluster 5.1 *Synechococcus* genera [3]. *Prochlorococcus* is the smallest known phototroph that inhabits subtropical, oligotrophic open ocean environments, where it thrives under extremely limiting concentrations of essential nutrients and contributes significantly to chlorophyll (Chl) biomass and primary production [4]. Marine *Synechococcus* spp. are better adapted to coastal water environments, although they do occur in the open ocean and their geographic distribution overlaps with that of *Prochlorococcus* [5]–[7]. On ribosomal RNA phylogeny *Prochlorococcus* and marine *Synechococcus* form a monophyletic clade [8]. The adaptations of *Prochlorococcus* and marine *Synechococcus* to their complementary ecological niches have been the subject of many investigations (e.g., [6], [7], [9]–[12]). There is emerging evidence that picocyanobacteria specifically may become even more implicated in the global carbon cycle with the escalating effects of climate change on the world's oceans. Ongoing climate warming has been correlated with a decline of total phytoplankton biomass [13], [14], and a shift towards a major dominance of picoplankton in phytoplankton communities is anticipated [15].

Among cyanobacteria *Prochlorococcus* and marine *Synechococcus* have the smallest genomes [4]. Minimization of a genome by selective reduction and deletion of non-essential genes is hypothesized to be an evolutionary strategy to decrease the amount of energy and nutrients needed to maintain cellular processes in a resource-limited environment [16]. Hence, reduced genome size may explain the ecological success of picocyanobacteria in oligotrophic areas of the ocean [1]. Comparative analyses of genomes of *Prochlorococcus* and marine *Synechococcus* reveal a substantial gene flow among *Prochlorococcus* and marine *Synechococcus* spp. [17], as well as the presence of strain-specific genomic islands [4]. Despite high similarity among their 16S rRNA gene sequences, the rest of their genomes are surprisingly divergent and exhibit variable GC content [18]. This divergence is likely due to *Prochlorococcus*' high mutation rates in combination with the lack of several DNA repair enzymes in the genome, as a direct result of the aggressive genome reduction [10]. A notable exception from the observed sequence divergence patterns is a protein-coding gene of unknown function, termed PSHCP for *Prochlorococcus*/*Synechococcus* Hyper Conserved Protein [18]. The PSHCP ORF encodes a polypeptide of 62 amino acid residues that exhibits 100% amino acid sequence conservation and is universally present in this clade of marine picocyanobacteria.

Such remarkable amino acid sequence conservation and consistent inclusion within the clade suggests that the PSHCP gene product performs an essential role in a picocyanobacterial cell. The DNA sequence of the ORF has accumulated numerous synonymous substitutions and its nucleotide composition mimics GC content of the host genome, indicating that the protein has been under purifying selection since the last common ancestor of the *Prochlorococcus* and marine *Synechococcus*, and has not been recently transferred among the members of the clade [18]. The gene synteny in the immediate genomic region surrounding the *PSHCP* gene is conserved across all examined picocyanobacteria: the gene is flanked on its 5′ end by *rpl19*, encoding the large ribosomal subunit protein 19 and the tryptophanyl-tRNA, and on its 3′ end by the aspartyl-tRNA and *gltX*, encoding a glutamyl tRNA synthetase (Fig. 2 in [18]). Since in prokaryotes the genes of functionally interacting proteins tend to be present in operons [19], the functional assignments of neighboring genes suggest that PSHCP could have a role in protein translation. Predicted features of the protein provide additional clues to its function. PSHCP is highly basic with a predicted isoelectric point (pI) of 11.3 and a high proportion (16%) of positively charged amino acids, suggestive of PSHCP's role in RNA/DNA-interactions [18].

At the time of initial discovery, PSHCP lacked homologs in any other cyanobacteria or other organisms for which sequence data was available in GenBank [18]. Presence of the PSHCP gene is now reported in four freshwater *Synechococcus* spp. strains isolated from Laurential Great Lakes and in a photosynthetic endosymbiont of the amoeba *Paulinella chromatophora* [20]. Similar to their marine counterparts, freshwater *Synechococcus* spp. dominate the euphotic zone of oligotrophic lakes, both in numbers and in overall productivity. In the freshwater *Synechococcus* spp. strains PSHCP retained its 100% amino acid sequence identity to the marine homologs and is flanked by the same genes in the genome [20]. Constitutive transcription of *PSHCP* in the freshwater strains was observed under various irradiances and under limiting conditions of nitrogen and phosphorus, and PCR experiments suggested that *rpl19* and *PSHCP* are co-transcribed [20]. In the highly reduced genome of the endosymbiont (chromatophore) of *Paulinella chromatophora*, the *PSHCP* protein is 89% identical to its picocyanobacterial homologs and is also flanked by *rpl19* and *gltX* genes [20]. Although it is no longer 100% conserved, it is remarkable, however, that the PSHCP gene is retained in the chromatophore genome that underwent a three fold size reduction from the genome of its ancestral free-living cyanobacterium, again pointing to the potentially essential role of PSHCP. The closest relative of chromatophore is a freshwater cyanobacterium from the *Cyanobium* clade, a sister clade to the clade of *Prochlorococcus* and marine *Synechococcus*, suggesting that PSHCP has originated in the common ancestor of marine and freshwater picocyanobacteria. In this study, we used a combination of bioinformatic and laboratory approaches to evaluate expression of the PSHCP gene, to quantify the PSHCP protein abundance, and to suggest possible PSHCP functions.

## Materials & Methods

### Promoter Prediction

Homologs of the PSHCP gene were retrieved from GenBank using BLAST [21] and *Synechococcus* sp. WH 8102 as a query (since PSHCP amino acid sequences are identical in all picocyanobacteria, the choice of strain will not affect the results). To predict potential promoters, WebLOGOs [22] were constructed for the DNA sequences of the region upstream of the PSHCP gene and compared with the consensus sequence for the experimentally determined −10 promoter element from *Prochlorococcus* sp. MED4 [23].

### Culturing

Cultures of *Synechococcus* sp. WH 8102 and *Prochlorococcus* spp. MED4 and MIT 9313 obtained from the Provasoli-Guillard National Center for Marine Algae and Microbiota were grown at 22°C in a 1∶4 dilution in PCR S11 and Pro99 medium, respectively [24]. MED4 was exposed to 40 µmol photons m^−2^s^−1^ of light, and WH 8102 and MIT 9313 were exposed to 20 µmol photons m^−2^s^−1^. Cultures were harvested at a quantum yield of photosynthesis between 0.4 and 0.6 determined by the ratio of the variable fluorescence (F_V_) to the maximum fluorescence (F_M_) using a Xenon-PAM fluorometer (Walz), as described previously [25].

### RT-PCR Data

**Figure 1:**
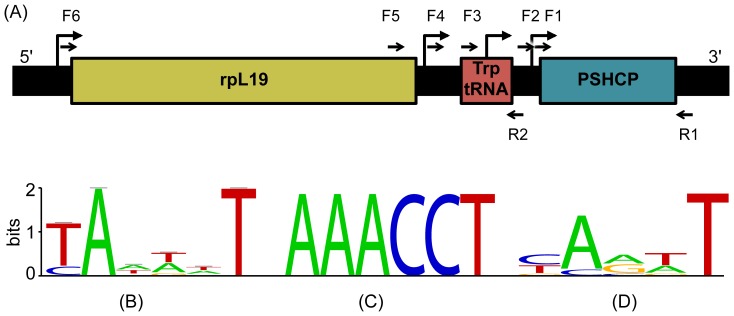
Schematic presentation of the genomic region around the PSHCP gene. Panel A. Mapping of promoters and primers onto the region. In all examined picocyanobacterial genomes the upstream region consists of the ribosomal protein L19 (rpL19) and tryptophanyl-tRNA genes. The locations of predicted promoters are shown by bent arrows, while the locations of the reverse (R1) and forward (F1 to F6) primers used for RT-Q-PCR analysis are indicated by half arrows (see Table S1 for further details). Panels B–D. Conservation of predicted and experimentally determined promoters across homologous regions from 18 *Prochlorococcus marinus* and *Synechococcus* spp. genomes. The regions were visualized using the WebLOGO web site (http://weblogo.berkeley.edu/). Panel B. Conservation of experimentally determined promoters from *Prochlorococcus marinus* MED4 [23]. Panel C. Conservation of predicted promoter located 37 bases into the *trp-tRNA* gene. Panel D. Conservation of the predicted promoter in the 5′ region 12–14 bases upstream of the *PSHCP* start codon. This promoter is present in all analyzed genomes except *Synechococcus* sp. RCC307. For promoters predicted upstream of the *trp-tRNA* and *rpl19* genes (panel A) WebLOGOs are not shown.

Cell pellets were obtained by centrifugation and RNA was extracted using the Trizol Kit (Life Technologies) as per the manufacturer's instructions. RNA extracts were treated with DNase I (Life Technologies) and quantified using the Quant-it RNA Broad Range Assay Kit (BR Kit, Life Technologies). Total RNA (60 ng) was reverse transcribed with random hexamer primers using the iScript cDNA Synthesis Kit (Bio-Rad), with reverse transcriptase omitted from the –RT negative control samples. Primers were designed to amplify specific regions surrounding the *pshcp* gene (Table S1 and Figure 1A). Calibrated standards were produced by amplifying the entire region of interest for all species (869 bp for WH 8102, 835 bp for MIT 9313, and 817 bp for MED4) using genomic DNA as a template. The PCR products were purified using a MinElute PCR Purification Kit (Qiagen) and quantified using the Quant-it DNA BR Kit (Life Technologies). Triplicate samples were prepared for RT-PCR using iQ SYBR Green Supermix (Bio-Rad) with a final cDNA content of 1.5 ng and 40 nM of forward and reverse primers. Samples were amplified using the following program in an iCycler thermal cycler (Bio-Rad): 95°C for 2 min; 40 cycles of 95°C for 30 s, 54°C for 30 s, 72°C for 30 s; 5 min at 72°C.

### Cloning of *pshcp* gene

Cell pellets were obtained from WH 8102 cell culture by centrifugation, and genomic DNA was extracted using the Ultraclean Microbial DNA Isolation Kit (MoBio). Primers were designed to specifically amplify the *pshcp* gene with incorporated restriction enzyme cut sites to facilitate downstream subcloning. The 199 bp *pshcp* insert was amplified using PCR, purified using a HiYield PCR DNA Extraction Kit (Real Genomics) and quantified as described above. The insert was cloned into the pCR2.1 vector and transformed into One Shot competent *E. coli* cells using the TOPO Cloning Kit (Life Technologies). Plasmid DNA was isolated from selected *E. coli* colonies using the Wizard Plus SV Miniprep DNA Purification System (Promega) and quantified as described above. The presence and integrity of the insert were confirmed by DNA sequencing by McGill University and Genome Quebec Innovation Centre.

The *pshcp* gene was subcloned into the pIVEX 2.4d vector following sequential restriction enzyme digestion by NcoI and XmaI (BioLabs), cleaning of the fragment (MinElute Gel Extraction Kit, Qiagen) and ligation with T4 DNA ligase reaction (Life Technologies) and transformation into competent DH5α *E. coli* cells.

### Overexpression, purification, and preparation of PSHCP for antibody production

The Rapid Translation System (RTS) 500 *E. coli* HY Kit (Roche) was used for large scale overexpression of the PSHCP protein for antibody production. Purified overexpressed PSHCP was separated by gel electrophoresis on 2-well 1.0 mm Novex 4–12% Bis-Tris NuPAGE gels (Life Technologies) and stained with SimplyBlue SafeStain (Life Technologies). Bands corresponding to PSHCP were excised and used as immunogen for antibody elicitation in rabbits (Agrisera AB).

### Antibody Validation

The specificity of the antibody was determined by comparing the immune serum to the pre-immune and by performing a competition assay, pre-incubating the immune serum with the immunogen protein to block reactivity (data not shown).

### Whole Cell Protein Extraction and Quantitative Immunoblotting

Cell pellets were harvested from 50 mL of each culture and resuspended in 200 µL of protein extraction buffer (1X AEBSF, Roche and 1X PSB, Agrisera). Samples were then homogenized using the CY:24×2 rotor of the FastPrep-24 Instrument (MPBIO) for two 1 min periods with 1 min of cooling on ice after each treatment. The protein extracts were then centrifuged at 10,000×g for 3 min to remove insoluble material and unbroken cells. Protein concentrations of extracts were quantified using the microplate DC protein assay (Bio-Rad) with bovine gamma globulin (BGG) as a comparative protein standard.

The sample preparation, SDS-PAGE, immunoblotting and quantitation protocols were followed according to those described previously [26]. Immunoblotting of PSHCP required a shorter transfer of 45 min. onto nitrocellulose membranes. Primary antibodies (Agrisera) were diluted in 2% ECL advance blocking agent (GE Healthcare) in TBS-T, RbcL 1∶10,000 and PSHCP 1∶2,000. HRP-conjugated goat anti-rabbit (Agrisera) was employed as the secondary antibody at a dilution of 1∶40,000. Immunoblots were developed with ECL Advance (GE Healthcare) and images were captured on a VersaDoc Imager (BioRad) and analyzed using Quantity One and Image Lab 3.0 software (BioRad).

### Pull Down Assay: Bait Preparation

Recombinant His_6_-PSHCP was overexpressed and purified with the NiNTA Purification System (Life Technologies) under native conditions, and employed as bait protein in the pull down assay. Genomic *pshcp* was PCR amplified using specific primers designed to facilitate its insertion into a pET303/C-terminal His_6_-tag vector, and the presence and proper sequence of the *pshcp* insert was subsequently confirmed as described above. The ligated pET303 vector was transformed into BL21 *E. coli* overexpression cells (Life Technologies). The overexpression of *pshcp* was induced with IPTG for 3 hr according to the expression guidelines, and cells were harvested by centrifugation.

The lysate was prepared under native conditions, according to the manufacturer's protocol for bacterial cell lysates with some alterations. Buffer concentrations of imidazole were increased in the lysate preparation (25 mM) and in the subsequent purification of His_6_-PSHCP in order to generate a more pure product. Lysis was achieved using a Branson Digital Sonifier at 70% amplification with 3×10 s, 3×35 s bursts on ice. The lysate was centrifuged at 13,000×g, for 15 min, at 4°C to remove cellular debris. The manufacturer's instructions for purification under native conditions were followed. The column was washed 2X with 35 mM imidazole native binding buffer (NBB), 2X with 50 mM, and eluted with 500 mM. 100 µg bait protein was brought to a final volume of 1–1.5 mL in 25 mM imidazole NBB using an Amicon 3K Ultra-15 Centrifugal Filter Unit.

### Pull Down Assay: Prey Preparation

A whole cell native extraction of *Prochlorococcus* and marine *Synechococcus* was performed on cell pellets harvested from 200 mL of culture of significant biomass (day 7 of growth) by centrifugation. The pellet was resuspended in a solution of 1X EBA (110 mM KOAc, 0.5% Triton X-100, 100 mM NaCl, 1 mM AEBSF, pH 7.4) with 0.05% Nonidet P-40 at a maximum total volume of 300 µL. The cells were subject to two flash-freeze-thaw (FT) cycles in liquid nitrogen to allow for partial breakage, and four subsequent rounds of sonication/FT, each round consisting of 4×10 s bursts on ice at 70% amplification followed by FT. The final solution was centrifuged at 10,000×g, for 5 min, at room temperature. The remaining pellet was re-treated with the same procedure in order to extract all possible protein. The amount of protein in the combined extracts was determined using the microplate DC protein assay with BGG used as a protein standard. A portion of the extract was diluted with 1X EBA to obtain 500 µg of protein in 500 µL (1 mg/mL).

### Pull Down Assay

NiNTA resin was resuspended and 50 µL each was placed into sterile 1.5 mL microcentrifuge tubes. Resins were equilibrated according to manufacturer's native purification protocol, using 500 µL sterile ddiH_2_O and 25 mM imidazole NBB. To experimental columns, prepared bait protein was added (100 µg in 1–1.5 mL). To control columns, an equal volume of 25 mM imidazole NBB was added. Tubes were rotated end-over-end for 1 hr at room temperature. The non-binding bait solutions were aspirated and 500 µg prepared prey sample was added to all tubes (1∶5 bait to prey mass ratio). Tubes were rotated end-over-end for 2 hr at room temperature to allow proteins to bind. The non-binding prey solutions were aspirated and resins were washed 3–4X with 25 mM imidazole NBB. Proteins were eluted in 5×100 µL fractions of 500 mM imidazole NBB.

### In-solution Tryptic Digest

The pull down products, experimental and control, were subject to an in-solution tryptic digest in preparation for mass spectrometry analysis. To each sample, TRIS (1 M), pH 8.3 was added to a final concentration of 50 mM. DTT was added to a final concentration of 10 mM and the samples were incubated at 50°C for 30 min. Iodoacetamide (1 M) was added to a final concentration of 40 mM. The samples were vortexed briefly and incubated in the dark at room temperature for 30 min. A 1 µg/µL trypsin solution was added at a trypsin: protein ratio of approximately 1∶40. The samples were incubated overnight (16–18 h) at 37°C. The reaction was stopped by adding 1/9^th^ volume of 1% TFA solution. Samples were frozen at −20°C until mass spectrometry analysis.

### Mass Spectrometry Analysis

Protein tryptic digests were analyzed by gradient nanoLC-MS/MS using a Quadrupole Orbitrap (Q-Exactive, Thermo-Fisher Scientific) mass spectrometer interfaced to a Proxeon Easy Nano-LC. Samples were adjusted to 1% aqueous acetic acid and injected (10 µL) onto a narrow bore (20 µm long ×100 µm inner diameter) C18 pre-column packed with 5 µm ReproSil-Pur resin (Thermo-Fisher Scientific). High resolution chromatographic separation was then achieved on a Thermo-Scientific Easy C18 analytical column with dimensions of 100 mm by 75 µm i.d. using 3 µm diameter ReproSil-Pur particles. Peptide elution was achieved using an acetonitrile/water gradient system, with LC-MS grade water and acetonitrile (VWR). Solvent A consisted of 0.1% formic acid (Sigma-Aldrich) in water and solvent B was made up of 90/9.9/0.1 acetonitrile/water/formic acid. A linear acetonitrile gradient was applied to the C18 column from 5–30% solvent B in 120 min followed by 100% B for 10 min at a flow rate of 300 nL/min.

The outlet diameter of the nano-flow emitter on the Q-Exactive (15 µm) was biased to +1.9 kV and positioned approximately 2 mm from the heated (250°C) transfer capillary. The S-lens of the mass spectrometer was maintained at 100 V. The Q-Exactive mass spectrometer was calibrated in positive ion mode with mass standards (caffeine, MRFA peptide and Ultramark) every three days as recommended by the instrument manufacturer. Mass spectrometric data was acquired in data dependent acquisition (DDA) mode, whereby a full mass scan from 350–1200 Th was followed by the acquisition of fragmentation spectra for the five most abundant precursor ions with intensities above a threshold of 20,000. Precursor ion spectra were collected at a resolution setting of 35,000 and an AGC (automatic gain control) value of 1×10^6^. Peptide fragmentation was performed using high energy collision induced dissociation in the HCD cell and MS/MS spectra were collected in the Orbitrap at a resolution of 17,500 and an AGC setting of 1×10^5^. Peptide precursors were selected using a repeat count of 2 and a dynamic exclusion period of 20 s.

Mass spectrometric protein identification data was analyzed using Proteome Discoverer version 1.3 (Thermo-Fisher Scientific) employing the Sequest scoring algorhithm. FASTA databases were obtained from Uniprot [27] for *Escherichia coli* (sp. BL21, 2041 kb), *Prochlorococcus marinus* (strains MED4, 774 kb and MIT 9313, 1028 kb) and *Synechococcus* sp. (strain WH 8102, 1010 kb). Searches were performed with the following settings: (a) enzyme specificity of trypsin with 2 allowed missed cleavages, (b) precursor and fragment tolerances were 10 ppm and 0.8 Da, respectively, (c) a variable modification of methionine oxidation (+15.99 Da), and (d) a fixed modification of cysteine carbamidomethylation (+57.02 Da). Proteome Discoverer 1.3 calculated a strict false discovery rate (FDR) of 0.1% based on the results of a decoy (reverse) database search. Peptides were scored with Sequest using minimal Xcorr values of 1.62 for +2 ions and 1.79 for +3 ions. Proteins were assigned a positive identification if at least one peptide was identified with high confidence or at least two unique peptides were identified with medium confidence.

### Survey of PSHCP presence in GenBank and draft cyanobacterial genomes

Using the PSHCP protein from *Prochlorococcus marinus* str. CCMP1986 as a query in BLASTP searches, homologs of the PSHCP were retrieved from GenBank and the IMG portal [28]. The homologs were examined for ORF length and for the presence of amino acid substitutions. Amino acid composition of the PSHCP and pI were predicted using the EMBOSS package [29]. Conserved domains were predicted using CD search [30].

### Information Content of Ribosomal RNA Genes in PSHCP-containing Cyanobacteria

110 completed genomes of free-living cyanobacteria available in GenBank were screened for the presence of PSHCP using BLAST searches and the PSHCP protein in *Prochlorococcus marinus* AS9601 as a query [21]. 5 S, 16 S and 23 S ribosomal RNA genes from these cyanobacterial isolates, as well as from *Escherichia coli* K-12 substr. MG1655 (used as a reference), were downloaded from the SILVA database [31] and aligned using the SINA program [32]. Alignments were edited manually for several mis-aligned regions and the alignment positions with>50% gaps were eliminated. The alignments were screened for “signature” positions, defined as a nucleotide in an alignment site present in ≥90% of PSHCP-containing cyanobacteria and in ≤10% of the remaining cyanobacteria (analogously to [33]). The variation in the alignment sites in cyanobacteria that have or do not have PSHCP was also summarized using Shannon entropy [34]. More specifically, frequencies *f* of each nucleotide *N* (*A, C, U* and *G*) at each site *s* of 23 S rRNA alignment were calculated for the two groups of cyanobacteria and uncertainty *H* at site *s* (in bits) was calculated using Shannon's formula [34]:
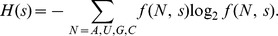



Total information content of the alignment site *T(s)* was calculated on a 0–1 scale, and then a gap penalty was applied:
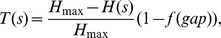
where *H_max_*  = 2 bits. Average information across all sites *T_avg_* was calculated for each group, to compensate for the fact that two cyanobacterial groups have different level of overall sequence divergence. The relative information for each site *R(s)* was defined as
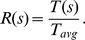



The *R(s)* values were mapped onto a secondary structure model of *Escherichia coli* described by Petrov *et al.* [35]. Distances of *E.coli*'s rRNA residues to the nearest amino acid residue of the ribosomal protein L2 were determined using the 3D structure information from the 3R8T record of the Protein Data Bank [36].

## Results

### PSHCP is universally present in one cyanobacterial clade

A survey of newly available cyanobacterial genomes reveals that PSHCP continues to be present in all *Prochlorococcus marinus* and marine *Synechococcus* genomes, as well as in freshwater strains of *Synechococcus* [20], in *Cyanobium* spp. and in the chromatophore genome of *Paulinella chromatophora* (Table S2). On an rRNA phylogenetic tree of cyanobacteria, these lineages form a monophyletic clade [3]. As noted in [18], the observed length variants of the *pshcp* genes are due to a misannotation of the start codon and a two amino acid variation at the C-terminus. Additionally, amino acid substitutions were observed in the PSHCP homologs from the genomes of *Synechococcus* sp. strains RCC307 and WH7803, and of the *Paulinella*'s chromatophore. The latter genome has undergone significant size reduction and exhibit an increased mutation rate [37], therefore it is not surprising that its PSHCP homolog does not show the extreme amino acid sequence conservation seen in the free-living cyanobacteria. Overall, the survey of the PSHCP homologs preserves our earlier observation that this protein is under strong purifying selection [18].

### Amino Acid Composition and Predicted Biochemical Properties of PSHCP

PSHCP has a predicted isoelectric point of 11.3 and contains 10 (16%) positively charged amino acids (8 arginines and 2 lysines). Moreover, PSHCP contains seven glycines. The predicted secondary structure consists of an alpha helix and four small beta strands (Fig. 3 in [18]). These properties are consistent with those of many ribosomal proteins, which have stretches of sequences rich in arginine and lysine, as well as high glycine content in the “extensions” [38]. A few ribosomal proteins contain SH3-like domains, and PSHCP has a weak similarity to an SH3-like domain, as detected in a Conserved Domain Database [39] search (data not shown). Tertiary structures of very small proteins (including those of several ribosomal proteins) are often stabilized by disulfide bonds or binding to zinc [40]; however, PSHCP's amino acid sequence lacks the indicative cysteines or histidine residues that would be required for Zn-binding fold to function properly.

### Transcriptional Analyses Identify Likely Transcription Start Sites for PSHCP Gene

To confirm PSHCP expression and to determine if PSHCP gene is expressed polycistronically with neighboring genes, we performed RT-Q-PCR experiments on *Prochlorococcus marinus* strains MED4 and MIT9313, and on marine *Synechococcus* sp. strain WH 8102. Utilizing the experimentally determined promoter sequence in *Prochlorococcus marinus* strain MED4 [23] (Figure 1B), we predicted promoters in the region upstream of the PSHCP genes (Figure 1C and D). To determine which, if any, of the predicted promoters are used to transcribe the *pshcp* gene, we performed RT-Q-PCR with a series of forward primers ranging from the beginning of the *pshcp* coding region through to the beginning of the *rpl19* gene. The inclusion of a standard curve allowed for absolute quantitation and ultimately a comparison between the strains. Table 1 presents the number of copies of each transcript per ng of RNA template. *Prochlorococcus marinus* strain MED4 has the highest number of copies of *pshcp* transcripts per ng of total RNA. In this strain, 42.8% of the transcripts appear to arise from the promoter predicted between the *tryptophanyl-tRNA* gene and the *pshcp* gene, whereas there is little to no apparent transcription from this promoter in the other strains. The bulk of the *pshcp* transcripts in MIT 9313 (74.9%) and WH 8102 (81.1%) strains arise from the strong predicted promoter within the *tryptophanyl-tRNA* gene. Larger transcripts are detected in all three strains, but to a greater degree in MED4, indicating some co-transcription with the *tryptophanyl-tRNA* and *rpl19*. It is possible that some of the shortest *pshcp* transcripts detected in MED4 arise from the polycistronic processing of mRNA rather than from transcription from the predicted promoter between *pshcp* and the *trp-tRNA*. Our results are largely in agreement with the earlier report [20] of co-transcription of *PSHCP* with *rpl19*, the *trp-tRNA* and the downstream *asp-tRNA*, but not with the downstream *gltX* gene in both the freshwater *Synechococcus* sp. strain ARC-11 and the marine *Synechococcus* strain WH 8102. We, however, measure the pool of long transcripts containing both *rpl19* and *pshcp* to represent less than 2% of the total transcript pool. Our transcriptional observations for the region near *pshcp* gene are in general consistent with findings of complex transcriptional patterns of bacterial genomes (e.g., [41], [42]).

**Table 1:** Quantitative Transcript Analysis.

RT-Q-PCR Product	Copies per ng of RNA template (% of the total *pshcp* transcripts)		
	MED4	MIT 9313	WH 8102
*hcp* only	2826 (42.8)	96 (5.4)	0 (0)
*hcp* +5′ region	629 (9.5)	1329 (74.9)	679 (81.1)
*hcp* + *Trp-tRNA*	1566 (23.7)	201 (11.3)	13 (1.6)
*hcp* + *Trp-tRNA* +5′ region	900 (13.6)	0 (0)	43 (5.1)
*hcp* + *Trp-tRNA* +3′ end *rpl19*	684 (10.4)	121 (6.8)	93 (11.1)
*hcp* + *Trp-tRNA* + *rpl19*	0 (0)	29 (1.6)	8 (1.0)
**Total**	6606	1776	837

Examination of the global gene expression patterns in *Prochlorococcus* MED4 and MIT 9313 show down-regulation of *pshcp* in both strains under nitrogen deprivation conditions [43] and down-regulation of *pshcp* in MIT 9313 (but not MED4) in response to phosphorus starvation [44]. Under both nitrogen and phosphorus limitation the transcription of *pshcp* changes in parallel with the expression of genes involved in protein translation [43], [44]. Constitutive expression of *pshcp* reported in [20] did not vary with light level or nutrient limitation. Since the latter study used a single end-point RT-PCR method to measure the level of transcription as opposed to RT-Q-PCR or microarray, subtle differences in expression such as those reported by Tolonen et al. [43] and Martiny et al. [44] may have gone undetected.

### PSHCP is a relatively abundant protein

The level of PSHCP protein in whole cell extracts of *Prochlorococcus* and marine *Synechococcus* cultures was on the order of that of the RuBisCO large subunit protein (RbcL), indicating that PSHCP is a relatively abundant protein (Table 2). No correlation was observed between the number of *pshcp* transcripts per ng total RNA and the fmoles of PSHCP protein per ug total cell protein in the cell extracts.

**Table 2:** Quantitative Protein Analysis.

Protein Detected (fmoles/ug total protein)	Complex	*Prochlorococcus* MED4	*Prochlorococcus* MIT 9313	*Synechococcus* WH 8102
RbcL	RuBisCO	59±15	20±8	71±8
PSHCP	unknown	19±8	26±9	8±1

### Potential Binding Partners of PSHCP

A large number of proteins were identified by the pull down assay (Table 3 and Table S3). However, three proteins (the 50S ribosomal protein Rpl2, the Photosystem I protein PsaD and the Ycf48-like protein) were pulled down from extracts of *all* strains in the presence of PSHCP, but few or none in its absence. Given that PSHCP is so conserved in all *Prochlorococcus* and marine *Synechococcus* sequenced to date, thus its essential function and binding partners should also be conserved across strains, we have focused our analysis on proteins that were pulled down only in the presence of PSHCP in all three strains examined. Both Rpl2 and PsaD have basic isoelectric points (11.1–11.6 and 7.0–8.7, respectively), and therefore their interactions with PSHCP unlikely to be artifactual consequences of non-specific electrostatic interactions between PSHCP and a prey protein. Since Rpl2 and PsaD would both be highly positively charged at physiological pH, they would also not be expected to interact non-specifically *in vivo*.

**Table 3:** Proteins of Unique Peptides Identified in Experimental Pull Down.

Protein Name	Accession Number	Strain	# of Unique Peptides	Molecular Weight (kDa)	Isoelectric point (pI)
**50 S Ribosomal Protein L2**	Q7U4J7	WH 8102	3	31.6–31.7	11.1–11.6
**50 S Ribosomal Protein L2**	Q7V539	MIT 9313	2	31.6–31.7	11.1–11.6
**50 S Ribosomal Protein L2**	Q7UZV0	MED4	8	31.6–31.7	11.1–11.6
**Photosystem I reaction center subunit PsaD**	Q7U4M3	WH 8102	1	15.7	7.0–8.7
**Photosystem I reaction center subunit PsaD**	Q7V564	MIT 9313	3	15.7	7.0–8.7
**Photosystem I reaction center subunit PsaD**	Q7UZS7	MED4	4	15.7	7.0–8.7
**Ycf48-like protein**	Q7U9P8	WH 8102	1	36.4–37.2	4.7–5.3
**Ycf48-like protein**	Q7V4Q3	MIT 9313	1	36.4–37.2	4.7–5.3
**Ycf48-like protein**	Q7V301	MED4	1	36.4–37.2	4.7–5.3

### Substitution patterns in the ribosomal RNA genes of PSHCP-containing cyanobacteria reveal non-compensatory substitutions

If PSHCP is interacting with Rpl2 protein (and hence likely be part of the ribosome), it may be recruited to stabilize ribosomal RNA structure. In the latter case, we would expect to observe non-compensatory substitutions in rRNA of PSHCP-containing cyanobacteria. Examination of 5 S, 16 S and 23 S rRNA nucleotide substitution patterns in 110 cyanobacterial isolates indeed revealed a few faster evolving sites in the 23 S rRNA of the PSHCP-containing cyanobacteria (Figure S1). Notable non-compensatory substitutions are located in the helical stems 18, 64, 75, 84 and 94 (Figure S1). Helical stems 75 and 76 are in proximity to the ribosomal protein L2 (Figure S2), with which PSHCP protein interacts (Table 3), suggesting that these helical stems could correspond to the regions of 23 S rRNA stabilized by the interactions of PSHCP with the RNA.

## Discussion & Conclusions

The modern ribosome is an RNA/protein complex that catalyzes protein synthesis in all living organisms. RNA molecules of the ribosome are catalytically active [45], while the role of the ribosomal proteins is to stabilize the structure of RNAs, often by “filling in the cracks between RNA helices” [38]. In light of these observations, it has been suggested that the primordial ribosome likely consisted of RNA alone and ribosomal proteins were recruited later. A few ribosomal proteins are universally present in the three domains of life and therefore were likely present already at the time of the Last Universal Common Ancestor (LUCA). Genes for these universal ribosomal proteins are usually clustered in the genomes into the universal ribosomal operons. More than half of the ribosomal proteins, however, are either domain-specific or patchily distributed across various taxonomic groups. Non-universal ribosomal proteins are likely newer additions to the ribosome [45], and genes for these ribosomal proteins are usually scattered across the genome.

Predicted features of the amino acid sequence of PSHCP and its observed interaction with the ribosomal protein Rpl2 favour a hypothesis that PSHCP is a group-specific ribosomal protein. It is not located within one of the universal r-protein operons, but it is encoded near Rpl19, a Bacteria-specific ribosomal protein, although it does not appear to be significantly co-transcribed with Rpl19 (see Table 1). Rpl2 is a ribosomal protein that plays a central role in the ribosome and is located near the peptidyl transferase center [45]. According to SH3-hunter [46], Rpl2 may interact with SH3-containing proteins. It is possible that PSHCP interacts with Rpl2 via its predicted SH3-like domain. Additionally, PSHCP may stabilize rRNA structure at positions where non-compensatory substitutions are observed.

Our pull-down assays also reveal an association of PSHCP with two proteins in the photosynthetic complex, which suggests a role linking synthesis of the photosystem subunits and their assembly into native complexes. The Ycf48-like protein has been shown to play a role in Photosystem II assembly in the freshwater cyanobacterium *Synechocystis* sp. strain PCC 6803 [47]. More recently Komenda *et al*. [48] demonstrated that the Psb27 assembly factor, which we found associated with PSHCP in the examined *Prochlorococcus* strains (Table S3), can interact with Photosystem I as well as Photosystem II under certain conditions. This is further suggestive of a role for PSHCP linking the ribosome to photosystem assembly.

Further structural and biochemical studies of PSHCP are needed to firmly establish the functional role of this protein in the picocyanobacterial cell. To further explore the association of PSHCP with the ribosome and/or the photosystems, picocyanobacterial ribosomes and photosystems should be isolated and probed with the PSHCP specific antibody reported here. Examination of the suggested protein-protein interactions in a bacterial two-hybrid system would determine whether PSHCP and these identified binding partners are indeed interacting in the cell. The evolutionary studies of the ribosome suggest that ribosomes have over time recruited ribosomal proteins [45]. If PSHCP's role as a ribosomal protein is confirmed, its further study may provide additional clues into the evolution of the ribosome.

## Supporting Information

Figure S1 **Nucleotide conservation of 23 S rRNA in PSHCP-containing cyanobacteria in comparison to other cyanobacteria.** The conservation is measured as a relative information content (see Materials & Methods for details). The difference in the relative information content between PSHCP-containing cyanobacteria and other cyanobacteria is color coded (see color bar) and mapped onto a predicted structure of 23 S rRNA of *Escherichia coli* [35]. Nucleotides on the red side of the spectrum correspond to the residues evolving faster in the PSHCP-containing cyanobacteria. Nucleotides shown in black correspond to sites that are absent in> 50% of cyanobacteria. Different domains of 23 S rRNA are designated by 0 and roman numerals I through VI. Helices are designated by numbers 1–101 in blue. Residue numbers (in brown) correspond to the nucleotides in *Escherichia coli* sequence.
(PDF)

Figure S2 **Visualization of possible interactions of the ribosomal protein L2 with 23 S rRNA.** Distances (in angstroms) of each nucleotide (P atom in the phosphate group) of the 23 S rRNA to the nearest residue (α-C atom of the amino acid) of the ribosomal protein L2 were calculated using crystal structure of the bacterial ribosome (PDB ID 3R8T). The distances were color coded (see scale bar on the figure) and mapped onto a predicted structure of 23 S rRNA of *Escherichia coli* [35]. Residues in black correspond to the nucleotides that were not resolved in the crystal structure. For notations of the 23 S rRNA features see legend to the Figure S1.
(PDF)

Table S1 **Nucleotide sequences of primers used in the RT-Q-PCR experiments.**
(PDF)

Table S2 **Presence of PSHCP homologs in genomes.**
(PDF)

Table S3 **Results of proteomics analysis from PSHCP pull down assay.** The spreadsheet contains the complete results of tandem mass spectrometric analysis of the pull down experiment with PSCHP. Analysis of each of the three strains is presented in a separate sheet (labeled with the strain name). Proteins discussed in the text are highlighted in green.
(XLSX)

### 

Column notations are as follows:

- *Column A:* UNIPROT accession number for the protein.
- *Column B:* Protein Score. The Sequest algorithm calculates a cross-correlation (Xcorr) value for every identified peptide. The protein score is the sum of Xcorr values for all peptides linked to a particular protein.
- *Column C:* Score for the control pull down (i.e. minus PCHCP). For definition of the score see description of column B.
- *Column D:* UNIPROT annotation of the protein.
- *Column E:* Coverage of the protein by the observed peptides.
- *Column F:* Number of unique peptides per protein.
- *Column G:* Molecular weight of the protein in kDa.
- *Column H:* Predicted isoelectric point of the protein.
