# Supplementary figures and images for: Quantitative and Functional Characterization of the Hyper-Conserved Protein of Prochlorococcus and Marine Synechococcus
Source: PLoS One. 2014 Oct 31;9(10):e109327. doi: 10.1371/journal.pone.0109327 (PMC4215834; doi:10.1371/journal.pone.0109327)

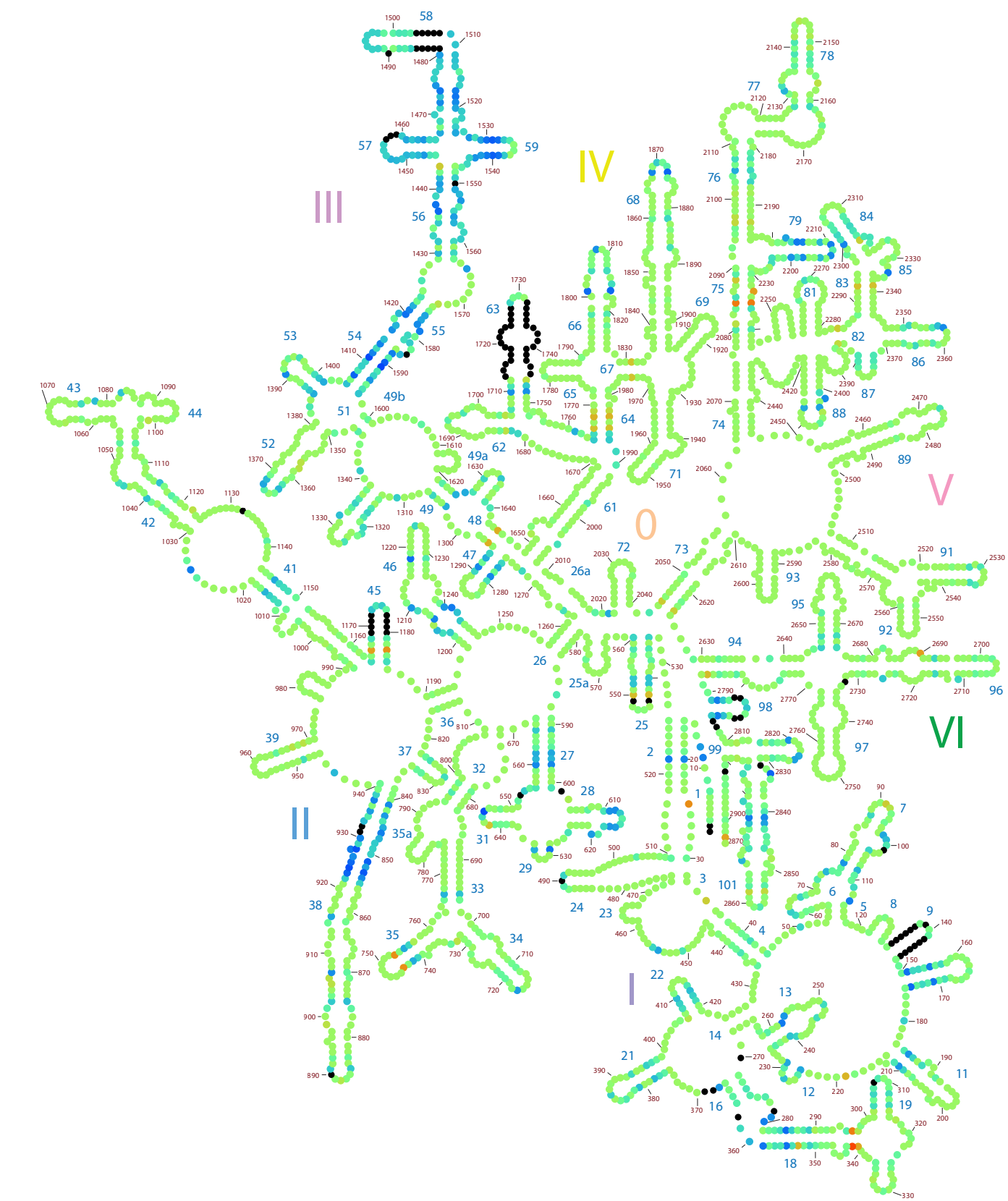

Relative Information Difference

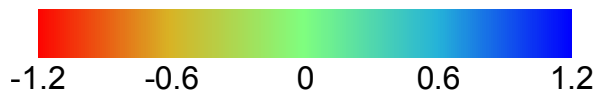

Supplement: Figure S1 — Nucleotide conservation of 23 S rRNA in PSHCP-containing cyanobacteria in comparison to other cyanobacteria. The conservation is measured as a relative information content (see Materials & Methods for details). The difference in the relative information content between PSHCP-containing cyanobacteria and other cyanobacteria is color coded (see color bar) and mapped onto a predicted structure of 23 S rRNA of Escherichia coli [35]. Nucleotides on the red side of the spectrum correspond to the residues evolving faster in the PSHCP-containing cyanobacteria. Nucleotides shown in black correspond to sites that are absent in> 50% of cyanobacteria. Different domains of 23 S rRNA are designated by 0 and roman numerals I through VI. Helices are designated by numbers 1–101 in blue. Residue numbers (in brown) correspond to the nucleotides in Escherichia coli sequence. (PDF) [file pone.0109327.s001.pdf]

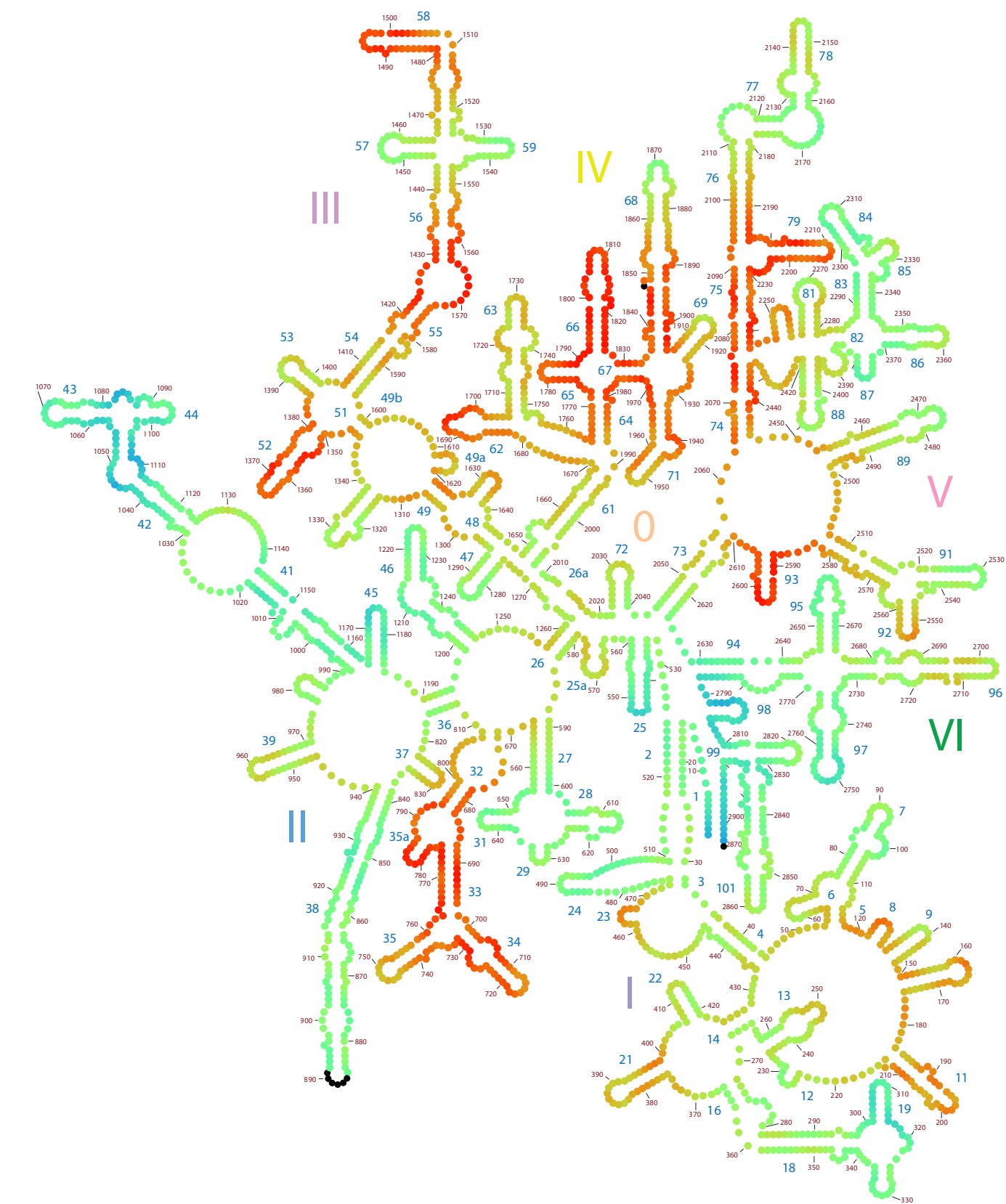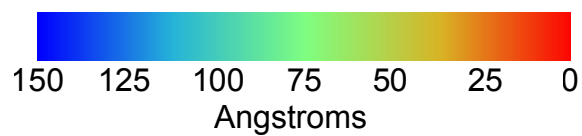

Supplement: Figure S2 — Visualization of possible interactions of the ribosomal protein L2 with 23 S rRNA. Distances (in angstroms) of each nucleotide (P atom in the phosphate group) of the 23 S rRNA to the nearest residue (α-C atom of the amino acid) of the ribosomal protein L2 were calculated using crystal structure of the bacterial ribosome (PDB ID 3R8T). The distances were color coded (see scale bar on the figure) and mapped onto a predicted structure of 23 S rRNA of Escherichia coli [35]. Residues in black correspond to the nucleotides that were not resolved in the crystal structure. For notations of the 23 S rRNA features see legend to the Figure S1. (PDF) [file pone.0109327.s002.pdf]
