# Supplementary material for: Quantitative and Functional Characterization of the Hyper-Conserved Protein of Prochlorococcus and Marine Synechococcus
Source: PLoS One. 2014 Oct 31;9(10):e109327. doi: 10.1371/journal.pone.0109327 (PMC4215834; doi:10.1371/journal.pone.0109327)
Supplement: Table S1 — Nucleotide sequences of primers used in the RT-Q-PCR experiments. (PDF) [file pone.0109327.s003.pdf]

Table S1. Nucleotide sequences of primers used in the RT-Q-PCR experiments.

Primer	MED4	MIT 9313	WH 8102
**F1**	GATGGAGTTAGATCTTCAACCTGG	GGTGATGTCGTCAAAGTGCTCG	CGGTTCTGAGCATGGAGTTGG
**F2**	CCACGGCGCGTTTGGATC	GGCGCGTCCGATGGC	CCGATGAACCCCGTTGTGTAG
**F3**	GCGTCGTTAGTTCAGTTGGTAG	GGCGCGTCCGATGGC	GCGCCGTTAGTTCAGTTGGTAG
**F4**	CAACCTAACGGTCATAAAGGCG	CTCGTTCAATCCAAATACCATCACC	GCCTCGTTCAATCAAGTCGC
**F5**	GAAAAGCTACTCGCGTAAAACAAC	GCTTTTCTATCTGCGGGAACGG	GCTTTTCTATCTGCGGGAACGG
**F6**	ATGGCAAAAGAGAAACAAGAAACTG	CGGAGCTGGGATGACGG	CGCGGAGCTGGGATGG
**R1**	GGCCTAAAGCCTGCTGGTTC	CCTGCGGGTTCGATTAGG C	CAGGGACGGAAGCCCGC
