# Supplementary material for: Quantitative and Functional Characterization of the Hyper-Conserved Protein of Prochlorococcus and Marine Synechococcus
Source: PLoS One. 2014 Oct 31;9(10):e109327. doi: 10.1371/journal.pone.0109327 (PMC4215834; doi:10.1371/journal.pone.0109327)
Supplement: Table S2 — Presence of PSHCP homologs in genomes. (PDF) [file pone.0109327.s004.pdf]

Table S2. Presence of PSHCP homologs in genomes.

Accession number	Locus Tag	Organism name	Length variation
**YP_007045635.1**	Cyagr_1114	*Cyanobium gracile* PCC 6307	62 a.a.
**EDY37537.1**	CPCC7001_416	*Cyanobium* sp. PCC 7001	MVPD+62 a.a.
**YP_002048693.1**	PCC_0022	*Paulinella chromatophora*	MSGVRVLHGAFKPSLAILS+ 62 a.a (89% identity)
**YP_001008924.1**	A9601_05291	*Prochlorococcus marinus* str. AS9601	62 a.a.+QK
**EEE40790.1**	P9202_1566	*Prochlorococcus marinus* str. MIT 9202	62 a.a.+QK
**YP_001550360.1**	P9211_04751	*Prochlorococcus marinus* str. MIT 9211	MSGVQVLHGAFDHPYSSFLVLR+62 a.a.
**YP_001483756.1**	P9215_05541	*Prochlorococcus marinus* str. MIT 9215	62 a.a.+QK
**YP_001090723.1**	P9301_04991	*Prochlorococcus marinus* str. MIT 9301	62 a.a.+QK
**YP_001016697.1**	P9303_06811	*Prochlorococcus marinus* str. MIT 9303	MSGVQVLHGASDGPSCSFLVPR+62 a.a.
**YP_396971.1**	PMT9312_0474	*Prochlorococcus marinus* str. MIT 9312	62 a.a.+QK
**NP_895135.1**	PMT1307	*Prochlorococcus marinus* str. MIT 9313	62 a.a.
**YP_001010854.1**	P9515_05381	*Prochlorococcus marinus* str. MIT 9515	62 a.a.
**YP_001014357.1**	NATL1_05301	*Prochlorococcus marinus* str. NATL1A	62 a.a.
**YP_292998.1**	PMN2A_1807	*Prochlorococcus marinus* str. NATL2A	62 a.a.
**NP_874865.1**	Pro0472	*Prochlorococcus marinus* subsp. marinus str. CCMP1375	62 a.a.
**NP_892592.1**	PMM0474	*Prochlorococcus marinus* subsp. pastoris str. CCMP1986	62 a.a.
**ACT10292.1**	n/a*	*Synechococcus* sp. ARC-11	62 a.a.
**ACT10294.1**	n/a*	*Synechococcus* sp. ARC-21	62 a.a.
**EAU70123.1**	BL107_10017	*Synechococcus* sp. BL107	62 a.a.
**N/A****	contig08658	*Synechococcus* sp. CB0101	62 a.a.
**N/A****	contig06975	*Synechococcus* sp. CB0205	62 a.a.
**YP_731366.1**	sync_2166	*Synechococcus* sp. CC9311	MSGVQVLHGASDDPLLAVFLISS+62 a.a.
**YP_380971.1**	Syncc9605_0642	*Synechococcus* sp. CC9605	MFPSCTFPVLS+62 a.a.
**N/A****	Syn9616DRAFT_ scaffold1.1	*Synechococcus* sp. CC9616	62 a.a.
**YP_377719.1**	Syncc9902_1717	*Synechococcus* sp. CC9902	62 a.a.
**ACT10299.1**	n/a*	*Synechococcus* sp. KD3a	62 a.a.
**ACT10297.1**	n/a*	*Synechococcus* sp. LS0504	62 a.a.
**YP_001227921.1**	SynRCC307_1665	*Synechococcus* sp. RCC307	62 a.a., R61→H61, P62→ N62
**EAU74499.1**	RS9916_33367	*Synechococcus* sp. RS9916	62 a.a.
**EAQ69597.1**	RS9917_09186	*Synechococcus* sp. RS9917	62 a.a.
**EAQ74693.1**	WH5701_13905	*Synechococcus* sp. WH 5701	MSGVQVLHGALDAPSCSFLVPD+62 a.a.
**YP_001225558.1**	SynWH7803_1835	*Synechococcus* sp. WH 7803	62 a.a., I55→N55
**EAR19986.1**	WH7805_13738	*Synechococcus* sp. WH 7805	62 a.a.
**N/A****	Syn8016_Contig50.1	*Synechococcus* sp. WH 8016	62 a.a.
**NP_897916.1**	SYNW1825	*Synechococcus* sp. WH 8102	62 a.a.
**EEX07732.1**	SH8109_2473	*Synechococcus* sp. WH 8109	62 a.a.

*Sequences are not from genome projects.

**Draft genome; BLAST search was performed at the IMG portal.
